# Satisfaction with Food: Profiles of Two-Parent Families with Adolescent Children

**DOI:** 10.3390/ijerph192416693

**Published:** 2022-12-12

**Authors:** Blanca Villalobos, Horacio Miranda, Berta Schnettler

**Affiliations:** 1 Doctorado en Ciencias Agroalimentarias y Medioambiente, Facultad de Ciencias Agropecuarias y Medioambiente, Universidad de La Frontera, Temuco 4780000, Chile; 2 Departamento de Ingeniería Química, Facultad de Ingeniería y Ciencias, Universidad de La Frontera, Temuco 4780000, Chile; 3 Departamento de Producción Agropecuaria, Facultad de Ciencias Agropecuarias y Medioambiente, Universidad de La Frontera, Temuco 4780000, Chile; 4 Scientific and Technological Bioresource Nucleus (BIOREN-UFRO), Universidad de La Frontera, Temuco 4780000, Chile

**Keywords:** satisfaction with food, eating habits, latent profiles

## Abstract

The objectives of this study were to distinguish family profiles based on the level of satisfaction with food-related life (SWFoL) of mothers, fathers, and adolescents, and to characterize the profiles based on the three family members’ diet quality and nutritional status, family’s eating habits, parental feeding practices, parent’s nutritional knowledge, and sociodemographic characteristics. Questionnaires were applied to a sample of 300 two-parent families with adolescent children, aged between 10 and 17 years, in Temuco, Chile. The questionnaires used were: satisfaction with food-related life (SWFoL); adapted healthy eating index (AHEI); family eating habits questionnaire (FEHQ); and the nutritional knowledge subscale. They also answered questions about eating habits and provided data to determine BMI and sociodemographic characteristics. Four different profiles were identified with respect to SWFoL: “Families satisfied with their food” (50.6%); “Fathers and mothers moderately satisfied with their food, children satisfied” (23.2%); “Families extremely satisfied with their food” (16.6%); and “Fathers and mothers satisfied with their food, children extremely dissatisfied” (9.7%). The profiles demonstrated heterogeneity in SWFoL. Higher levels of SWFoL (mothers, fathers and adolescents) were associated with healthier eating habits. These results contribute to new studies that enable understanding of how families’ healthy eating habits are part of improving quality of life.

## 1. Introduction

Subjective well-being (SWB) as part of quality of life (in its subjective dimension) expresses the degree of satisfaction people have with specific or global aspects of their existence, where positive moods prevail [1]. In this context, satisfaction with life (SWL) is a cognitive component of SWB and is defined as the positive valuation a person makes of their life in general or of specific domains (for example, family, studies, work, health, friends, free time) [2,3,4]. Since food is an essential component for good health and a high quality of life [5], there is increasing interest in the literature to address the relationship between SWB and food consumption [6,7,8]. Food, as a specific domain of life, can be valued according to satisfaction with food-related life (SWFoL), which refers to a person’s assessment of their food and eating habits [9]. Different studies have determined that a high SWFoL is positively associated with SWL in adolescents and adults [10,11,12,13,14].

Different studies have reported that SWL and SWFoL are heterogenous in adults [15,16,17] and adolescents [17,18,19,20], which has been linked to different eating behaviors, nutritional states and sociodemographic characteristics. Although there are studies that have addressed the identification of profiles based on parental feeding practices, family eating habits, satisfaction with family life, satisfaction with life, and others [21,22,23,24], there is still a paucity of studies that have focused on identifying profiles on the basis of SWFoL in the different members of family groups, and distinguishing the differences in their eating habits, bearing in mind the heterogeneity that exists in SWFoL, an aspect to which the present study endeavors to contribute.

Studying SWFoL levels in different family members at the same time makes it possible to understand the various influences that exist in families [17]. The links between SWFoL and eating habits, nutritional knowledge, sociodemographic characteristics and nutritional status contribute to the knowledge of what the quality of life is like for people and, in particular, two-parent families with adolescent children.

From this background, this work examines whether there are profiles of two-parent families with adolescent children that differ according to the members’ level of satisfaction with food, their eating habits, diet quality, nutritional status, parental feeding practices, nutritional knowledge and sociodemographic characteristics. Therefore, the objectives of this study were: (a) to distinguish family profiles based on the level of satisfaction with food-related life (SWFoL) of mothers, fathers, and adolescents; and (b) to characterize the profiles based on the three family members’ diet quality and nutritional status, family’s eating habits, parental feeding practices, parents’ nutritional knowledge, and sociodemographic characteristics.

### Literature Review

Several studies have established that the highest levels of SWFoL are associated with healthy eating habits in adults [17,21,25,26] and adolescents [17,27]. This suggests that people believe food contributes directly to their health [9,28]. However, in 2016 more than 1.9 billion people 18 years or older were overweight, of which, more than 650 million were obese, and more than 340 million children and adolescents (from 5 to 19 years) were overweight or obese [29]. In the case of Chile, 30% of the population is obese, ranking second in the region of Latin America and the Caribbean [30]. Particularly in the population from 15 to 24 years, 17.1% present obesity [31]. Obesity in children (fifth grade elementary) is 27.7%, and in adolescents (first year high school) it is 14.7% [32]. There is a myriad of causes of the increase in overweight and obesity, among which is the change in eating habits, with an increase in the availability of ultra-processed foods and a reduction in traditional cooking preparations, which use fresh and healthy foods [30].

On this basis, the contribution of a healthy diet and the promotion of good eating habits among adolescents and young people [33] for suitable growth and development in adolescents have been recognized [34]. In particular, parents act as agents of change because they play a fundamental role in the formation of the family eating environment, which is affected at different stages of the children’s development (from childhood to adolescence) [35]. Nevertheless, studies about what mechanisms at the family level influence diet quality in adolescents with overweight or obesity are quite limited because most studies have concentrated on small children [35]. Moreover, information on the quality of children’s diet in relation to the eating attitudes of their parents or caregivers remains undeveloped [36].

Nutritional knowledge and the parents’ eating behavior represent relevant variables that contribute to the eating preferences of their children and their eating patterns [37]. Parents who motivate and model healthy eating are more likely to have children who eat healthy foods [38]. In this sense, it has been found, on the one hand, that nutritional knowledge is influenced by people’s sociodemographic characteristics, and the mother having high nutritional knowledge is associated with the children having a better-quality diet [39]. On the other hand, the parents’ eating behavior is associated with parental feeding practices [40], which are specific techniques or behaviors that tend to be used to facilitate or limit food intake [41]. These are divided into specific sub-constructions; for example, coercive control considers the pressure to eat, and structured parental feeding considers “rules and limits”, whereas support or the promotion of autonomy includes nutritional education, negotiation, and others [42]. This is reflected, for example, in a greater consumption of fruits and/or vegetables among the children of parents who adopt practices of stimulus feeding, access and availability. By contrast, more restrictive or controlling practices are associated with unhealthy eating habits, such as the desire for and consumption of restricted foods, if these are available [35], affecting the child’s health and weight later in life [43], eating disorders, and lower levels of SWFoL [26].

For an individual analysis, the promotion of healthy eating needs to be differentiated by age, gender and nutritional status [44]. Some authors report that gender affects the perception of the healthy nature of food [45,46]. For example, [47] determined that young women have healthier eating habits than older women. On the other hand, nutritional state measured through the body mass index (BMI) could also be a variable related to feeding styles, the attitudes and behaviors that parents have in approaching their children’s eating, since it forms the children’s ability to self-regulate food intake [48,49,50]. It has been demonstrated that having more regular eating habits, healthier food choices and sufficient energy intake are BMI-related aspects [51]. In adolescents, BMI is related to the control of parental feeding practices used at family meals [21].

The impact of parental practices is reflected in the eating habits and subjective well-being of, for example, mothers and their children, which can also differ between single-parent and two-parent homes [13]. In this sense, it has been found that parents who motivate and model healthy eating are more likely to have children who eat healthy foods [38]. In addition, it must be considered that parental feeding practices not only affect the children’s current eating habits but will also influence how they will choose their own foods in the long term [42], considering that parental feeding practices correlate significantly with the BMI of adults and SWFoL [26]. Accordingly, it is relevant to understand the family mechanisms that can influence children’s food, which involves generating effective intervention strategies to control the dietary outcomes of adolescents [35].

On the other hand, the frequency of family meals is associated with children’s healthier food consumption [52]. There is an inverse relation between the frequency of family meals and body weight in parents and children. This relation seems to be applied to gender, region, ethnic group and socioeconomic status (SES) [53]. This last variable, in particular, has been related to the selection of healthy foods, finding that people with a high SES prefer or can access healthy foods more easily than people with a low SES [54,55,56].

## 2. Materials and Methods

### 2.1. Sample and Procedure

This study had a non-experimental and cross-sectional correlational design. The study used non-probability convenience sampling, forming a sample of 300 two-parent families with adolescent children aged between 10 and 17 years. Table 1 presents the characteristics of the families who participated in the study. The average number of family members was 4.4, with an average of 2.4 children. The average age of the adolescents interviewed was 13.2 years; 51.3% were male. With respect to the education of the family head, 50.3% had secondary education, 35.7% university education, 10.0% elementary education and 4.0% postgraduate. In relation to socioeconomic level, 35.0% were at a lower-middle level.

The families were recruited in seven schools where students from different socioeconomic levels attend. The coordination for the selection of the students was achieved through collaboration with the principal in each of the schools. The parents were contacted to advise them of the objectives of the study and inform them of the confidential handling of the data collected. The instruments were applied in the homes of the participating families, after an informed consent was signed by the parents and children. Each family member was interviewed individually, anonymously, and without the presence of the other family members. Data were collected between June and December 2016.

This study was part of a wider investigation from a research project whose general aim was “To evaluate eating habits and subjective well-being in Chilean families”. The project was approved by the Scientific Ethics Committee of the Universidad de La Frontera (File N°011/2016 of 30 March 2016).

### 2.2. Measures

Data were collected using the following instruments, which were answered by all the members of the families:

- Satisfaction with food-related life scale (SWFoL): Instrument developed by [9] which assesses a person’s valuation of their food and eating habits. It consists of five items grouped into a single dimension. The Spanish version of SWFoL was used [57], which has demonstrated adequate internal consistency in previous studies in Chile [17,58,59,60]. The respondents were asked to indicate their degree in agreement with each item using a 6-point Likert-type scale (1: Completely disagree; 2: Mostly disagree; 3: Slightly disagree; 4: Slightly agree; 5: Mostly agree; 6: Completely agree). SWFoL scores were calculated as the sum of the five scale items. Higher scores indicate greater satisfaction with food. In this study, SWFoL showed good internal consistency (Cronbach’s α: mothers = 0.857; fathers = 0.766; children = 0.910).

- Adapted healthy eating index (AHEI): Instrument adapted for Spanish-speaking populations by [61] based on the “*Healthy Eating Index*” developed by [62]. The participants were asked about their frequency of consumption for nine food groups: (1) Cereals and derivatives; (2) Vegetables; (3) Fruits; (4) Milk and dairy products; (5) Meat; (6) Vegetables; (7) Sausages and cold cuts; (8) Sweets; (9) Sugary drinks. According to the degree of compliance with nutritional recommendations, and according to the definition of food groups and the scoring criteria by [61], each group obtained a score from 0 to 10 points. In addition, a variable referring to dietary variety was assessed, for which 2 points are added if the person fulfills each of the daily recommendations and 1 point if they fulfill each of the weekly recommendations. The maximum score on the AHEI is 100 points, which was calculated by adding the scores obtained in each of the variables. Scores over 80 indicate a healthy diet; scores between 51 and 80 correspond to a diet that requires changes, and scores below 50 correspond to unhealthy diets [62].

- Family eating habits questionnaire (FEHQ): Instrument proposed by [63] that contains 14 items to assess how the respondents perceive their family’s eating habits (for example: My family eats large meals; In my family we make healthy meals; Food is an important part of my family life; Family members pressure me to eat even when I am not hungry). The participants responded to each item on a 5-point Likert-type scale (1: Never; 2: Occasionally; 3: Sometimes; 4: Often; 5: Always). The Spanish version of the FEHQ validated in Chile [20] was used. This version was applied in a previous study on adolescents and three dimensions were obtained: food portions; cohesiveness of family eating; and pressure to eat [21]. In [58] it was applied to a sample of mothers, fathers and adolescents, where it was adjusted to two factors. The first factor corresponded to “family meals and healthy eating”, made up of seven items, and the second factor was labeled “portions and pressure to eat” made up of five items. In this study, both factors obtained acceptable levels of internal consistency (Cronbach’s α: Factor 1, mothers = 0.723; fathers = 0.760; adolescents = 0.718; Factor 2, mothers = 0.758; fathers = 0.771; adolescents = 0.732). 

The following instrument was answered by mothers and fathers:

Nutritional knowledge: This corresponds to a subscale of the instrument “*Scale of parental knowledge of nutrition guidelines*” proposed by [64], which measures the respondent’s knowledge of the perception of food consumption recommendations by health experts. The subscale corresponds to a measurement of the consumption of nutrients, with response options: more; almost the same; less; try to avoid them; and I am not sure [65]. The answers were coded with code 1 for correct ones and code 0 for incorrect ones. In this study, the nutritional knowledge showed an acceptable internal consistency (Cronbach’s α: mothers = 0.711, fathers = 0.763).

The following instruments was answered only by the mothers:

Frequency of family meals [66]: This consists of the number of days in a week where the family meets to eat together (breakfast, lunch, supper and dinner). The response options range from 1 to 7 days.

To ascertain the families’ nutritional status and to determine BMI (kg/m^2^), mothers, fathers and adolescents were asked about their own weight and approximate height. The BMI was calculated for mothers and fathers according to World Health Organization criteria [67]. In the case of adolescents, the BMI was calculated according to indicators of the Chilean Ministry of Health [31].

Finally, to collect sociodemographic data in order to characterize the sample, each member of the family was asked about their age, only mothers and fathers were asked about their ethnic origin, and the mothers were asked about the number of members in the family, the number of children and the gender of the household head. To determine socioeconomic level according to [68], the education level and occupation of the family head was used.

### 2.3. Statistical Analysis

To measure the reliability of each scale, Cronbach’s alpha coefficient was used. A two-step process was used to distinguish and characterize the profiles based on the scores of SWFoL of fathers, mothers and adolescent children. In the first step, the family profiles were distinguished using a latent profile analysis (LPA) for continuous variables. This type of analysis makes it possible to investigate heterogeneity by segmentation into groups with similar perceptions [69]. The LPA models were tested iteratively, from one to seven clusters for the models of the LPA using the scores from SWFoL of the three family members. The best model was selected using the Bayesian information criterion (BIC) and the consistent Akaike information criterion (CAIC). To determine the number of latent profiles, the lowest BIC and CAIC were used, since these values indicate the optimal number of profiles [70].

After the three family members were grouped into the four profiles based on their SWFoL scores, the second step of the analysis involved characterizing these profiles based on statistical differences found in the variables concurrent with those scores. An analysis of variance (ANOVA) was used to determine differences among the profiles in the continuous variables. A test of homogeneity (Levene statistic) was performed. The variables that demonstrated homogenous variances (*p* ≤ 0.001) were subjected to the Tukey’s multiple comparison test. The variables with non-homogenous variances were subjected to Dunnett’s T3 multiple comparison test. In the case of the FEHQ, ANDEVA was conducted using the z-scores of the two factors obtained through factor analysis [20]. For the characterization of the profiles according to discrete variables, contingency tables and the Pearson Chi^2^ statistic were used. For the analysis, the SPSS software version 23.0 in Spanish for Windows was used. The LPA was applied using the Latent Gold 5.1 statistics software (Statistical Innovations Inc., Belmont, MA, USA).

## 3. Results

### 3.1. Latent Profiles According to Satisfaction with Food-Related Life

The LPA was performed with a series of 1–7 clusters applied to the SWFoL scores of the mothers, fathers and adolescents. The 4-cluster model showed the lowest values for BIC (5292.74) and CAIC (5319.74) (Table 2).

Table 3 provides the significance of the SWFoL scores of each family member.

**Figure 1:**
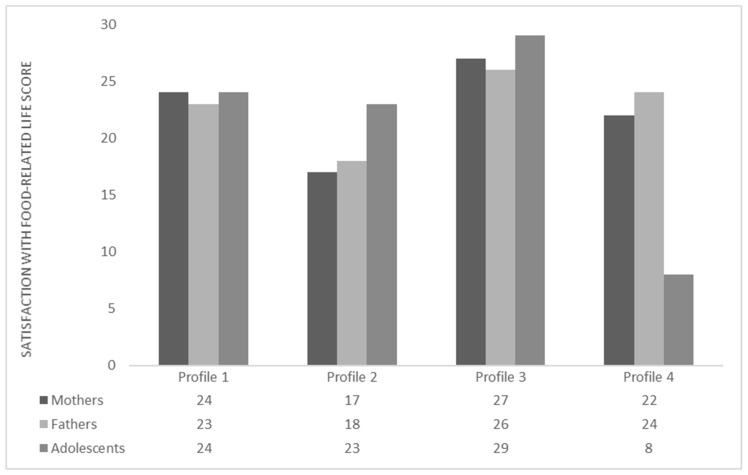
Profiles of two-parent families with adolescent children based on satisfaction with food-related life. Scores for satisfaction with food-related life were subjected to Dunnett’s T3 multiple comparisons test.

For the description of the profiles according to the scores obtained for each family member (mothers, fathers and adolescents) (Figure 1), the scores of the SWFoL were classified according to: 5–10 = Extremely dissatisfied; 11–15 = Dissatisfied; 16–20 = Moderately satisfied; 21–25 = Satisfied; 26–30 = Extremely satisfied [71]. The profiles obtained were: Profile 1—families satisfied with their food; Profile 2—fathers and mothers moderately satisfied with their food, children satisfied; Profile 3—families extremely satisfied with their food; Profile 4—fathers and mothers satisfied with their food, children extremely dissatisfied.

### 3.2. Description of the Latent Profiles

Next, the results obtained by profiles and their relationship to each analyzed variable are presented (Figure 1, Table 4 and Table 5).

Profile 1: Families satisfied with their food. This profile represented the greatest percentage of the sample (50.6%) (Table 4). Mothers (F = 88.680; *p* < 0.001) and fathers (F = 55.165; *p* < 0.001) in this profile obtained significantly higher average SWFoL scores than Profile 2, without differing statistically from the other profiles. The adolescents in Profile 1 had a significantly lower average SWFoL score than those in Profile 3 (F = 265.838; *p* < 0.001), but higher than in Profile 4 (Figure 1). The mothers (*p* < 0.001) and adolescents (*p* < 0.05) in this profile obtained statistically lower average AHEI scores than Profile 3. The fathers (*p* < 0.001) did not differ from the rest of the profiles. The families of this profile presented significantly average breakfast (*p* < 0.01) and lunch (*p* < 0.05) values than Profile 3. For the first factor on the FEHQ “Cohesiveness of family meals and healthy eating”, mothers (*p* < 0.001), fathers (*p* < 0.001) and adolescents (*p* < 0.001) presented statistically lower average z values than Profile 3 (Table 4).

Profile 2: Fathers and mothers moderately satisfied with their food, children satisfied. This profile represented 23.2% of the total sample (Table 4). The mothers and parents of this profile obtained average SWFoL scores significantly lower than the other profiles. The adolescents presented a significantly lower average SWFoL score than the adolescents in Profile 3, but higher than those in Profile 4 (Figure 1). The mothers and fathers of this profile presented the lowest average AHEI scores, although they did not differ from the values obtained by the mothers and fathers in Profile 1. The adolescents in Profile 2 also presented the lowest average AHEI score; however, it was only statistically lower than the average obtained by the adolescents in Profile 3. The families in this profile presented lower average breakfast and lunch values than the other profiles, but statistically lower than the average breakfast and lunch values in profile 3. In the first factor on the FEHQ, mothers, fathers and adolescents presented lower average z scores than the other profiles, although they did not differ from Profile 1 (Table 4).

Profile 3: Families extremely satisfied with their food. These families represented 16.6% of the total sample (Table 4). The mothers and adolescents in this profile obtained significantly higher average scores on the SWFoL than the rest of the profiles. The fathers in this profile had a significantly higher average SWFoL score than the fathers in Profiles 1 and 2, although they did not differ from Profile 4 (Figure 1). Mothers and adolescents presented the highest average AHEI scores, being statistically higher than Profiles 1 and 2. The fathers (*p* < 0.001) in Profile 3 obtained significantly higher average AHEI scores than Profile 2. The families in this profile obtained higher average breakfast and lunch values than the rest of the profiles; however, they were statistically higher than the average breakfast and lunch values in Profiles 1 and 2. In the first factor on the FEHQ in this profile, the families presented a higher average z value than the other profiles. Mothers and fathers obtained significantly higher average z values than Profiles 1 and 2, whereas the adolescents differed from the rest of the profiles (Table 4). The adolescents in this profile presented a significantly greater proportion with a BMI corresponding to malnourished (*p* < 0.01) and obese (*p* < 0.01) than the rest of the profiles (Table 5).

Profile 4: Fathers and mothers satisfied with their food, children extremely dissatisfied. With 9.7%, this was the profile with the lowest percentage of the total sample (Table 4). The adolescents in this profile obtained a significantly lower average SWFoL score than the rest of the profiles. Mothers and fathers presented a statistically higher average SWFoL score than in Profile 2. Mothers and fathers in Profile 4 had significantly higher average SWFoL scores than the mothers and fathers in Profile 2 (Figure 1). The fathers in this profile had a higher average AHEI score than the other profiles, differing from Profile 2. The mothers had a statistically higher AHEI score than the mothers in Profile 2. The adolescents did not differ from the other profiles in their average AHEI scores. The families in Profile 4 did not differ from the rest of the profiles in the average breakfast and lunch values. In the first factor on the FEHQ for this profile, mothers and fathers presented statistically higher average z values than Profile 2. The adolescents presented a statistically lower average z value than Profile 3 (Table 4). Profile 4 had a significantly greater presence of fathers of Mapuche origin (*p* < 0.05) (Table 5). The adolescents in this profile presented a significantly greater proportion with a low-weight BMI than the other profiles (Table 5).

It should be noted that, for the frequency of consumption of the nine food groups included in the AHEI, significant differences were only observed in the food groups “vegetables” and “sweets”, in mothers (*p* < 0.001) and fathers (*p* <0.001). In adolescents there were no significant differences in any of the nine food groups (*p* > 0.1) (Table 6). The mothers and fathers in Profile 3 presented higher percentages of daily consumption of vegetables, 86% and 74%, respectively, and a higher percentage in the “sweets” food group in the consumption frequency “never or almost never” (mothers, 50%; fathers, 44%). No significant differences were observed between profiles in nutritional knowledge were found between mothers and fathers, in the z scores of the second factor “food portions and pressure to eat” on the FEHQ, in the nutritional status of fathers and mothers and in the frequency of supper and dinner as a family (*p* > 0.1). With respect to the sociodemographic characteristics, no significant differences were observed (*p* > 0.1) in the socioeconomic level (SES), education level of the household head, age of each member of the families or the gender of the adolescents.

## 4. Discussion

The present study sought to distinguish family profiles based on the level of satisfaction with food-related life (SWFoL) of mothers, fathers, and adolescents, and to characterize the profiles based on the three family members’ diet quality and nutritional status, family’s eating habits, parental feeding practices, parent’s nutritional knowledge, and sociodemographic characteristics.

Using a latent profile analysis, four profiles of two-parent families were identified according to their level of SWFoL. The first profile corresponds to families satisfied with their food (50.6%). The second profile corresponds to fathers and mothers moderately satisfied with their food, and children satisfied (23.2%). The third profile represents families satisfied with their food (16.6%) and the fourth profile corresponds to fathers and mothers satisfied with their food, but children extremely dissatisfied (9.7%). The results showed that there are different profiles of families in relation to their members’ satisfaction with their food. The profiles presented heterogeneity in SWFoL scores between mothers, parents, adolescents and families, which is consistent with what has been indicated in other studies where SWFoL was heterogenous in adults and young people [15,16,72] and in families with adolescent children [17].

Generally, Profile 3 (mothers, fathers and adolescents) was the one that presented the highest average scores in the frequency of family meals, and cohesiveness of family meals, both variables being linked to high average SWFoL scores. For the variable AHEI, the scores obtained for each of the members of the families were different in the four profiles: mothers and adolescents in Profile 3 presented the highest average AHEI scores as well as the highest levels of SWFoL. This is in line with the quality of the diet being directly related to SWFoL in adults and adolescents [59,73,74,75,76,77].

In contrast, in Profile 2 the three members of the families presented the lowest average scores in the variables frequency of family meals, cohesiveness of family meals and AHEI. However, only mothers and fathers in this profile presented average SWFoL scores lower than the rest of the profiles.

The families felt more satisfied with their food when they got together for all the daily meals and, in particular, breakfast; moreover, in adults and adolescents there was a significant and positive association between shared family meals and SWFoL [78]. This was confirmed in our study because Profile 3 was the one with the greatest frequency of family meals (breakfast and lunch), as well as the highest average SWFoL score. In addition, there is a positive association between a greater frequency of family meals and healthier diets in adolescents [27,79]. However, Profile 2 was the one that presented the fewest number of days where the families ate together, both breakfast and lunch, and with a lower average SWFoL score.

In the frequency of consumption of the nine food groups included in the AHEI, significant differences were observed only in the “vegetables” and “sweets” food groups, in mothers (*p* < 0.001) and fathers (*p* < 0.001). The mothers and fathers in Profile 3 presented higher percentages in daily consumption of vegetables, 86% and 74%, respectively, and a greater percentage of sweets consumption in the frequency “never or almost never” (mothers, 50%; fathers, 44%). This profile is the one that presents the highest SWFoL and better eating habits in general. According to [52], eating habits, both of parents and children, have been positively associated with better food consumption patterns (greater consumption of fruits and vegetables, and a lower consumption of sweets). In our study, this occurred only in the case of mothers and fathers and in the consumption of vegetables and sweets.

It is worth noting that mothers obtained higher AHEI scores than fathers in the four profiles. This is similar to the report by [80], where the women obtained higher AHEI scores than the men. This could also be associated with women with low levels of well-being having worse diet qualities than those with higher levels of well-being [81]. The types of food consumed in families can affect food consumption in adolescents because the foods commonly consumed at each meal vary according to the local culture [82]. Nevertheless, the AHEI scores obtained by each member of the families and in each of the four identified profiles indicated that all require changes to be made to their diet.

In the cohesiveness of family meals and healthy eating, the three members of the families in Profile 3 also obtained higher values and the highest SWFoL score. This might be indicative of high SWFoL levels in adolescents being related to healthy eating habits and good family relations [14], and of parents being a model of healthy eating habits [83,84,85]. However, Profile 3 was the one that presented the highest percentage of adolescents with a BMI of obesity (14.0%) and malnourishment (18.0%), which would denote that frequent and cohesive family meals are not sufficient to achieve diets and healthier weights in adolescents [21]. Parental feeding practices adapt to different needs and demands according to a child’s growth and maturity [86]. There are also profiles of parents who, to influence their children’s eating, use multiple parenting practices in terms of feeding, either simultaneously or only a few practices, which can have positive and negative effects on their children [87].

This could also explain why Profile 4, where fathers were satisfied with their food, but children were extremely dissatisfied with theirs (lower average SWFoL score than the rest of the profiles) is the profile with adolescents with the highest percentage of low-weight BMI (26.7%), which would negatively affect the adolescent’s health, such as having an unhealthy weight, eating disorders and lower levels of SWFoL [26]. Adolescence requires a well-balanced diet, because it is a period of strenuous growth due to a greater amount of activity and the development of physical and cognitive functions [88].

Nutritional knowledge, BMI and SES were not statistically significant when related to SWFoL. Finally, it was not possible to relate some parental feeding practices, nutritional knowledge and the knowledge of foods to SWFoL, nor to determine the relation between SWFoL and sociodemographic characteristics.

### Limitations of the Study

This study has limitations that must be addressed in future investigations, such as its cross-sectional nature, which cannot measure causality, considering also that the information on weight and height was self-reported by the individuals in the sample. Moreover, it was a relatively small non-probability sample focused on a particular city in Chile. In addition, not being a longitudinal study, it was not possible to do a follow-up of the study participants, considering the COVID-19 pandemic of the last two years, which has very likely brought about changes in diets and eating habits, for example. The authors of [89] indicate that depression in itself could affect eating habits and lead to excessive eating and to weight increase. This was reflected by the COVID-19 pandemic and stay-at-home restrictions imposed, which generated an increase in food consumption and a change in food choices, which led to unhealthy diets. In different countries, an increase in unhealthy food consumption was observed, such as chocolate, canned foods, potato chips and fast food. Accordingly, there was also an increase in the obesity rate in adolescents. Quarantine and social distancing caused adolescents to also make changes in their lifestyle and eating habits [90].

Another aspect that could be addressed is an analysis of mothers and fathers in two-parent families with respect to parenting practices of feeding, nutritional knowledge, and eating habits, as well as differences in the eating habits between adolescent boys and girls.

Future studies should include the foods that adolescents consume during their school day, because families lack this knowledge [91]. It should also be investigated whether SWFoL profiles identified are consistent with other populations in order to better understand the contribution of parental practices and eating habits in families. It is also proposed that SWFoL-based profiles should be studied in single-parent families, since in this study only two-parent families were included. According to the current context, in light of high foreign migration into the country, how cultural differences influence families’ SWFoL and eating habits could be explored. Other sociodemographic variables such as parents’ occupations, children’s education and the family environment during meals could be studied to determine their relationship to SWFoL.

## 5. Conclusions

This study identified four profiles of two-parent families with adolescent children according to SWFoL levels. The profiles demonstrated the heterogeneity of SWFoL. Higher levels of SWFoL (mothers, fathers and adolescents) were associated with healthier eating habits. In diet quality, the AHEI scores obtained for each member of the families and in each of the four identified profiles indicated that all require changes in their diet. The frequency of meals and cohesiveness of family meals and healthy eating are relevant to adolescents obtaining diets and healthy weights. The results contribute to new studies that enable understanding how families’ healthy eating habits are part of improving the quality of life.

## Figures and Tables

**Table 1:** Sample characteristics (*n* = 300).

Characteristic	Total Sample
Mother’s age [Mean (*SD*)]	41.6 (6.8)
Father’s age [Mean (*SD*)]	44.1 (7.2)
Number of family members [Mean (*SD*)]	4.4 (1.0)
Number of children [Mean (*SD*)]	2.4 (1.0)
Interviewed adolescent’s age [Mean (*SD*)]	13.2 (2.3)
Interviewed adolescent’s gender (%)	
Female	48.7
Male	51.3
Breadwinner education (%)	
Elementary	10.0
Secondary	50.3
University	35.7
Postgraduate	4.0
Socioeconomic status (%)	
High and upper-middle	17.0
Middle	18.7
Lower-Middle	35.0
Low	24.0
Very low	5.3
Ethnic origin mother (%)	
Mapuche	19.4
Not Mapuche	80.6
Ethnic origin father (%)	
Mapuche	14.9
Not Mapuche	85.1
Satisfaction with food-related life [Mean (*SD*)]	
Mother	22.8 (4.7)
Father	22.9 (4.5)
Adolescent	22.8 (6.2)
AHEI [Mean (*SD*)]	
Mothers	64.5 (14.3)
Fathers	58.9 (14.6)
Adolescents	62.0 (14.9)
Number of days a week family eat together [Mean (*SD*)]	
Breakfast	4.2 (2.8)
Lunch	4.4 (2.6)
Supper	6.1 (1.9)
Dinner	3.9 (3.2)
FEHQ [Mean (*SD*)]	
Cohesiveness of family meals and healthy eating	
Mothers	4.2 (0.6)
Fathers	4.1 (0.6)
Adolescents	4.0 (0.6)
Food portions and pressure to eat	
Mothers	2.3 (0.9)
Fathers	2.4 (0.9)
Adolescents	2.4(0.9)
Nutritional knowledge [Mean (*SD*)]	
Mothers	8.3 (2.1)
Fathers	7.9 (2.4)
Mothers BMI (%)	
Normal range	24.0
Overweight	42.7
Obesity	33.3
Fathers BMI (%)	
Normal range	17.7
Overweight	55.7
Obesity	26.7
Adolescents BMI (%)	
Undernourished	6.3
Underweight	13.7
Normal range	53.7
Overweight	19.7
Obesity	6.7

**Table 2:** Summary of latent profile cluster models.

	LL	BIC(LL)	CAIC(LL)	Npar	Classification Error
1-Cluster	−2738.55	5511.32	5517.32	6	0.0000
2-Cluster	−2638.90	5351.96	5364.96	13	0.1007
3-Cluster	−2603.15	5320.37	5340.37	20	0.0932
4-Cluster	−2569.37	5292.74	5319.74	27	0.1237
5-Cluster	−2559.59	5313.11	5347.11	34	0.1876
6-Cluster	−2545.42	5324.69	5372.94	41	0.1967
7-Cluster	−2530.95	5335.69	5390.15	48	0.1754

LL = Log-likelihood; BIC(LL) = Bayesian information criterion based on the log-likelihood. CAIC(LL) = Consistent Akaike’s Information Criterion. Npar = Number of parameters.

**Table 3:** Significance of the indicators for the four profiles.

	Robust Wald Statistic	*p*-Value	R^2^
Mother SWFoL	86.84	1.00 × 10^−18^	0.3888
Father SWFoL	60.24	5.20 × 10^−13^	0.3093
Adolescent SWFoL	1566.82	1.9 × 10^−339^	0.7057

**Table 4:** Differences between the four latent classes’ profiles according to satisfaction with food-related life (SWFoL), adapted healthy eating index (AHEI), family meal frequency, family habits questionnaire (FEHQ) and nutritional knowledge.

	Profile 1	Profile 2	Profile 3	Profile 4	F	*p*-Value
Profile size	0.5055	0.2322	0.1655	0.0968		
AHEI						
Mothers ^2^	63.00 bc	61.17 c	70.45 a	69.30 ab	5.916	0.001
Fathers ^2^	58.02 ab	54.69 b	62.64 a	65.17 a	4.946	0.002
Adolescents ^2^	60.71 b	60.41 b	67.91 a	61.92 ab	3.286	0.021
Number of days a week family eat together						
Breakfast ^1^	4.04 b	3.47 b	5.32 a	4.23 ab	4.146	0.007
Lunch ^2^	4.31 b	3.81 b	5.40 a	4.53 ab	3.743	0.012
Supper ^1^	6.20	5.62	6.42	5.90	1.974	0.118
Dinner ^2^	3.86	4.22	3.74	3.67	0.295	0.829
FEHQ						
Cohesiveness of family meals and healthy eating						
Mothers ^2^	−0.04 bc	−0.39 c	0.47 a	0.19 ab	7.551	0.000
Fathers ^2^	−0.09 bc	−0.38 c	0.52 a	0.36 ab	9.839	0.000
Adolescents ^2^	−0.11 b	−0.32 b	0.72 a	0.03 b	12.787	0.000
Food portions and pressure to eat						
Mothers ^2^	−0.07	−0.00	0.18	0.13	1.011	0.388
Fathers ^2^	−0.04	−0.05	0.15	0.08	0.587	0.624
Adolescents ^1^	−0.12	0.07	0.29	0.03	2.293	0.078
Nutritional knowledge						
Mothers ^2^	8.25	8.29	8.40	8.37	0.073	0.975
Fathers ^1^	8.06	7.19	8.12	8.23	2.273	0.080

^1^ Different letters in the line indicate significant differences according to Dunnett’s T3 multiple comparisons test. ^2^ Different letters in the line indicate significant differences according to Tukey’s multiple comparisons test. Profile 1—Families satisfied with their food; Profile 2—Fathers and mothers moderately satisfied with their food, children satisfied; Profile 3—Families extremely satisfied with their food; Profile 4—Fathers and mothers satisfied with their food, children extremely dissatisfied.

**Table 5:** Differences (%) of the satisfied between the four latent profiles according to fathers’ ethnic origin and body mass indexes (BMI) of adolescents.

	Profile 1	Profile 2	Profile 3	Profile 4
Fathers’ ethnic origin (%)	*p* = 0.031			
Mapuche	11.4	12.5	16.7	33.3
Not Mapuche	88.6	87.5	83.3	66.7
Adolescents BMI (%)	*p* = 0.002			
Undernourished	3.7	5.2	18.0	3.3
Underweight	15.4	10.3	4.0	26.7
Normal range	54.3	58.6	54.0	40.0
Overweight	20.4	22.4	10.0	26.7
Obesity	6.2	3.4	14.0	3.3

*p*-value corresponds to the (bilateral) asymptotic significance obtained in Pearson’s Chi-square Test. Profile 1: Families satisfied with their food; Profile 2: Fathers and mothers moderately satisfied with their food, children satisfied; Profile 3: Families extremely satisfied with their food; Profile 4: Fathers and mothers satisfied with their food, children extremely dissatisfied.

**Table 6:** Differences (%) between the four latent profile classes the consumption frequency of the foods included in the adapted healthy eating index (AHEI) according for mothers, fathers and adolescents.

	Profile 1	Profile 2	Profile 3	Profile 4
Mothers Cereals and derivatives (%)	*p* = 0.356			
Daily consumption	16.7	19.0	32.0	23.3
Three or more times a week, but not daily	24.1	19.0	18.0	13.3
Once or twice a week	31.5	29.3	26.0	36.7
Less than once a week	9.9	19.0	6.0	6.7
Never or almost never	17.9	13.8	18.0	20.0
Mothers Vegetables (%)	*p* = 0.003			
Daily consumption	54.3	44.8	86.0	63.3
Three or more times a week, but not daily	32.1	37.9	12.0	23.3
Once or twice a week	12.3	13.8	0.0	13.3
Less than once a week	1.2	3.4	2.0	0.0
Never or almost never	0.0	0.0	0.0	0.0
Mothers Fruit (%)	*p* = 0.304			
Daily consumption	40.1	43.1	58.0	56.7
Three or more times a week, but not daily	34.6	22.4	28.0	26.7
Once or twice a week	17.3	25.9	10.0	6.7
Less than once a week	6.2	6.9	4.0	6.7
Never or almost never	1.9	1.7	0.0	3.3
Mothers Milk and dairy products (%)	*p* = 0.939			
Daily consumption	32.7	31.0	44.0	40.0
Three or more times a week, but not daily	29.0	25.9	30.0	23.3
Once or twice a week	22.2	25.9	16.0	23.3
Less than once a week	9.3	8.6	6.0	10.0
Never or almost never	6.8	8.6	4.0	3.3
Mothers Meat (%)	*p* = 0.108			
Daily consumption	17.9	12.1	10.0	6.7
Three or more times a week, but not daily	40.7	23.0	54.0	46.7
Once or twice a week	30.2	34.5	28.0	40.0
Less than once a week	9.9	13.8	2.0	6.7
Never or almost never	1.2	0.0	6.0	0.0
Mothers Legumes (%)	*p* = 0.149			
Daily consumption	1.9	0.0	2.0	3.3
Three or more times a week, but not daily	14.8	10.3	16.0	3.3
Once or twice a week	55.6	48.3	62.0	73.3
Less than once a week	17.9	27.6	20.0	16.7
Never or almost never	9.9	13.8	0.0	3.3
Mothers Sausages and cold meats (%)	*p* = 0.987			
Daily consumption	12.3	15.5	10.0	13.3
Three or more times a week, but not daily	22.2	22.4	24.0	13.3
Once or twice a week	22.2	25.9	24.0	20.0
Less than once a week	22.8	20.7	22.0	26.7
Never or almost never	20.4	15.5	20.0	26.7
Mothers Sweets (%)	*p* = 0.001			
Daily consumption	9.9	3.4	8.0	0.0
Three or more times a week, but not daily	19.1	22.4	14.0	20.0
Once or twice a week	27.2	37.9	6.0	23.3
Less than once a week	23.5	20.7	22.0	20.0
Never or almost never	20.4	15.5	50.0	36.7
Mothers Soft drinks with sugar (%)	*p* = 0.881			
Daily consumption	21.0	22.4	26.0	23.3
Three or more times a week, but not daily	14.8	10.3	18.0	10.0
Once or twice a week	18.5	25.9	12.0	16.7
Less than once a week	18.5	15.5	14.0	13.3
Never or almost never	27.2	25.9	30.0	36.7
Fathers Cereals and derivatives (%)	*p* = 0.223			
Daily consumption	15.4	13.8	20.0	16.7
Three or more times a week, but not daily	15.4	24.1	22.0	36.7
Once or twice a week	26.5	22.4	16.0	6.7
Less than once a week	14.8	8.6	18.0	10.0
Never or almost never	27.8	31.0	24.0	30.0
Fathers Vegetables (%)	*p* = 0.001			
Daily consumption	46.9	31.0	74.0	60.0
Three or more times a week, but not daily	32.1	34.5	22.0	36.7
Once or twice a week	17.9	25.9	4.0	3.3
Less than once a week	3.1	6.9	0.0	0.0
Never or almost never	0.0	1.7	0.0	0.0
Fathers Fruit (%)	*p* = 0.196			
Daily consumption	22.8	20.7	46.0	27.6
Three or more times a week, but not daily	42.0	34.5	28.0	41.4
Once or twice a week	22.2	25.9	18.0	20.7
Less than once a week	10.5	12.1	6.0	6.9
Never or almost never	2.5	6.9	2.0	3.4
Fathers Milk and dairy products (%)	*p* = 0.224			
Daily consumption	29.0	20.7	28.0	36.7
Three or more times a week, but not daily	19.8	22.4	34.0	20.0
Once or twice a week	28.4	22.4	4.0	16.7
Less than once a week	14.2	22.4	4.0	16.7
Never or almost never	8.6	13.8	8.0	10.0
Fathers Meat (%)	*p* = 0.664			
Daily consumption	18.5	19.0	12.0	30.0
Three or more times a week, but not daily	43.2	43.1	44.0	20.0
Once or twice a week	30.9	32.8	36.0	40.0
Less than once a week	4.9	5.2	6.0	6.7
Never or almost never	2.5	0.0	2.0	3.3
Fathers Legumes (%)	*p* = 0.460			
Daily consumption	1.9	1.7	4.0	3.3
Three or more times a week, but not daily	11.1	8.6	6.0	10.0
Once or twice a week	58.6	50.0	68.0	70.0
Less than once a week	19.1	20.7	16.0	13.3
Never or almost never	9.3	19.0	6.0	3.3
Fathers Sausages and cold meats (%)	*p* = 0.553			
Daily consumption	17.9	13.8	24.0	26.7
Three or more times a week, but not daily	24.1	24.1	26.0	16.7
Once or twice a week	24.1	27.6	26.0	16.7
Less than once a week	24.1	24.1	16.0	16.7
Never or almost never	9.9	10.3	8.0	23.3
Fathers Sweets (%)	*p* = 0.036			
Daily consumption	8.0	8.6	14.0	10.0
Three or more times a week, but not daily	17.9	19.0	14.0	13.3
Once or twice a week	21.0	25.9	10.0	6.7
Less than once a week	32.7	20.7	18.0	33.3
Never or almost never	20.4	25.9	44.0	36.7
Fathers Soft drinks with sugar (%)	*p* = 0.548			
Daily consumption	26.5	27.6	36.0	33.3
Three or more times a week, but not daily	24.1	17.2	18.0	13.3
Once or twice a week	16.0	19.0	8.0	16.7
Less than once a week	14.2	22.4	12.0	20.0
Never or almost never	19.1	13.8	26.0	16.7
Adolescents Cereals and derivatives (%)	*p* = 0.443			
Daily consumption	25.9	27.6	42.0	30.0
Three or more times a week, but not daily	27.8	20.7	30.0	26.7
Once or twice a week	28.4	29.3	10.0	23.3
Less than once a week	6.2	8.6	4.0	10.0
Never or almost never	11.7	13.8	14.0	10.0
Adolescents Vegetables (%)	*p* = 0.216			
Daily consumption	37.7	41.4	64.0	40.0
Three or more times a week, but not daily	37.7	34.5	22.0	26.7
Once or twice a week	18.5	15.5	10.0	26.7
Less than once a week	3.1	1.7	2.0	3.3
Never or almost never	3.1	6.9	2.0	3.3
Adolescents Fruit (%)	*p* = 0.105			
Daily consumption	31.5	29.3	58.0	46.7
Three or more times a week, but not daily	31.5	32.8	22.0	23.3
Once or twice a week	26.5	25.9	14.0	23.3
Less than once a week	8.6	6.9	6.0	6.7
Never or almost never	1.9	5.2	0.0	0.0
Adolescents Milk and dairy products (%)	*p* = 0.572			
Daily consumption	57.4	62.1	62.0	50.0
Three or more times a week, but not daily	19.1	12.1	16.0	26.7
Once or twice a week	12.3	15.5	12.0	16.7
Less than once a week	6.2	3.4	10.0	6.7
Never or almost never	4.9	6.9	0.0	0.0
Adolescents Meat (%)	*p* = 0.812			
Daily consumption	18.5	17.2	16.0	13.3
Three or more times a week, but not daily	33.3	41.4	42.0	43.3
Once or twice a week	35.8	34.5	34.0	26.7
Less than once a week	8.6	5.2	4.0	6.7
Never or almost never	3.7	1.7	4.0	10.0
Adolescents Legumes (%)	*p* = 0.065			
Daily consumption	1.2	5.2	2.0	10.0
Three or more times a week, but not daily	16.7	10.3	12.0	20.0
Once or twice a week	51.2	60.3	70.0	46.7
Less than once a week	20.4	10.3	14.0	16.7
Never or almost never	10.5	13.8	2.0	6.7
Adolescents Sausages and cold meats (%)	*p* = 0.631			
Daily consumption	9.3	8.6	12.0	13.3
Three or more times a week, but not daily	25.9	41.4	22.0	20.0
Once or twice a week	28.4	25.9	28.0	23.3
Less than once a week	20.4	10.3	22.0	23.3
Never or almost never	16.0	13.8	16.0	20.0
Adolescents Sweets (%)	*p* = 0.069			
Daily consumption	19.1	13.8	22.0	16.7
Three or more times a week, but not daily	30.2	27.6	12.0	20.0
Once or twice a week	25.3	22.4	24.0	26.7
Less than once a week	13.6	22.4	18.0	33.3
Never or almost never	11.7	13.8	24.0	3.3
Adolescents Soft drinks with sugar (%)	*p* = 0.362			
Daily consumption	28.4	29.3	40.0	26.7
Three or more times a week, but not daily	21.0	27.6	16.0	13.3
Once or twice a week	22.8	13.8	10.0	30.0
Less than once a week	12.3	19.0	16.0	16.7
Never or almost never	15.4	10.3	18.0	13.3

*p*-value corresponds to the (bilateral) asymptotic significance obtained in Pearson’s chi-square test. Profile 1—families satisfied with their food; Profile 2—fathers and mothers moderately satisfied with their food, children satisfied; Profile 3—families extremely satisfied with their food—Profile 4—fathers and mothers satisfied with their food, children extremely dissatisfied.

## Data Availability

Not applicable.
